# Challenge for One Health: Co-Circulation of Zoonotic H5N1 and H9N2 Avian Influenza Viruses in Egypt

**DOI:** 10.3390/v10030121

**Published:** 2018-03-09

**Authors:** Shin-Hee Kim

**Affiliations:** VA-MD Regional College of Veterinary Medicine, University of Maryland, College Park, MD 20742, USA; shinkim@umd.edu; Tel.: +1-301-314-4107; Fax: +1-301-314-6855

**Keywords:** zoonotic avian influenza virus, H5N1, H9N2, Egypt, vaccines

## Abstract

Highly pathogenic avian influenza (HPAI) H5N1 viruses are currently endemic in poultry in Egypt. Eradication of the viruses has been unsuccessful due to improper application of vaccine-based control strategies among other preventive measures. The viruses have evolved rapidly with increased bird-to-human transmission efficacy, thus affecting both animal and public health. Subsequent spread of potentially zoonotic low pathogenic avian influenza (LPAI) H9N2 in poultry has also hindered efficient control of avian influenza. The H5N1 viruses acquired enhanced bird-to-human transmissibility by (1) altering amino acids in hemagglutinin (HA) that enable binding affinity to human-type receptors, (2) loss of the glycosylation site and 130 loop in the HA protein and (3) mutation of E627K in the PB2 protein to enhance viral replication in mammalian hosts. The receptor binding site of HA of Egyptian H9N2 viruses has been shown to contain the Q234L substitution along with a H191 mutation, which can increase human-like receptor specificity. Therefore, co-circulation of H5N1 and H9N2 viruses in poultry farming and live bird markets has increased the risk of human exposure, resulting in complication of the epidemiological situation and raising a concern for potential emergence of a new influenza A virus pandemic. For efficient control of infection and transmission, the efficacy of vaccine and vaccination needs to be improved with a comprehensive control strategy, including enhanced biosecurity, education, surveillance, rapid diagnosis and culling of infected poultry.

## 1. Introduction

The avian influenza virus (AIV) belongs to the family *Orthomyxoviridae* and the genus *Influenza virus* A [1]. The virus has a negative-sense, single-stranded and segmented RNA genome and contains eight gene segments encoding at least 10 proteins: polymerase basic 1 (PB1), PB2, polymerase acid (PA), hemagglutinin (HA), nucleoprotein (NP), neuraminidase (NA), matrix 1 (M), M2, nonstructural (NS1) and NS2. The HA and NA proteins are surface glycoproteins and important for virus infectivity. The HA protein is responsible for virus attachment to the host cell and the major target of the humoral immune responses. The NA protein plays a role in the increase and spread of progeny virions by removing sialic acid from glycoproteins. 

The natural reservoirs of the virus are wild aquatic birds, with ducks, gulls and shorebirds being the primary hosts, which has contributed to the wide geographic spread and distribution of circulating viruses [2]. On the basis of antigenic specificity, 16 HA types and nine NA types have been detected in viruses isolated from wild waterfowl. Most AIVs cause little to no disease in aquatic birds. These low pathogenic avian influenza viruses (LPAIVs) contain an HA cleavage site, which can only be cleaved by proteases available in the intestinal and respiratory tract [3]. In contrast, highly pathogenic avian influenza viruses (HPAIVs) cause systemic infection and high mortality in chickens and other terrestrial poultry; and in ducks and other waterfowl depending on the strain of HPAIVs. Two subtypes (H5 and H7) of LPAIVs can naturally switch to a highly pathogenic phenotype by the spontaneous acquisition of a multibasic cleavage site during circulation in poultry [4,5,6]. The host range of *Influenza virus* A is mainly determined by the affinity of HA binding to terminal sialic acids of glycoproteins on the surfaces of respiratory epithelial cells [7]. Avian and human viruses preferentially bind to sialic acid linked to galactose via an α2-3 linkage (SAα2,3Gal) and galactose via an α2-6 linkage (SAα2,6Gal), respectively. The receptor binding site (RBS) is located at the membrane distal end of each HA monomer, and critical amino acids for host determinant are residues 222 and 224 in the HA of H5 (equivalent to residues 226 and 228 in the HA of H2 and H3) [8]. Specifically, HAs with Gln-222 and Gly-224 (avian and equine viruses) recognize SAα2,3Gal, whereas those with Leu-226 and Ser-228 (human viruses) recognize SAα2,6Gal. In addition, major antigenic drift of avian H5 has been found in proximity to the HA RBS with key mutations in the H3-corresponding antigenic A and B sites [9]. The two sites contain functional epitopes with high neutralizing efficiency. Antigenic matching between the vaccine and currently circulating field strains is critical for the efficacy of AIV vaccine [10,11,12,13].

In particular, the Asian-origin strain A/goose/Guangdong/1/96 (Gs/GD) lineage of H5 subtype HPAIV has become established in aquatic poultry as a reservoir and widespread across Asia, Europe, Africa and North America [2]. The virus has become genetically diversified represented by multiple phylogenetic lineages, classified as clades 0–9 [14]. Currently, only viruses from clades 0, 1 and 2 have infected humans since the initial 1997 outbreaks (Table 1) [15,16,17,18]. LPAI influenza A virus H9N2 was first isolated in the United States in 1966 in turkeys and was subsequently found to be prevalent among waterfowl [19]. Since the l990s, H9N2 viruses have spread widely among gallinaceous poultry in East and Central Asia, the Middle East and Africa. The North America and Eurasian lineages of H9N2 viruses are circulating in poultry and wild birds [20]. The Eurasian lineage harbors six genetically-distinct clades: G1-like, Y280-like, G9-like, BJ94-like, Y439-like and Korean-like clades (Table 2) [21,22]. The G1-like clade is further diversified into A, B, C and D subclades [19]. Groups A and B are circulating extensively in the Middle Eastern countries and have been identified since 1999. Inter-subtype reassortments have been detected between the co-circulating H9N2 and the highly pathogenic H5N1 or H7N3 viruses. Interestingly, G1-like H9N2 has been involved in generating reassortant viruses by donating their six internal genes to multiple novel genotypes of AIV, such as HPAI H5N1 [23,24,25], zoonotic H7N9 [26,27,28] and LPAI H10N8 [29].

Despite control effort, the virus remains endemic in poultry in China, Indonesia, Vietnam and Egypt and continues to cause outbreaks in poultry, as well as sporadic human infection [30]. Specifically, the cumulated number of confirmed human cases of H5N1 infection reported to the World Health Organization (WHO) to date is 860 with 454 fatal cases [31]. Egypt is one of the countries with the highest number of human fatalities due to zoonotic H5N1 infections. In 2011, H9N2 viruses were detected to be co-circulating and co-infecting the same hosts with H5N1 viruses [32] and subsequently have established an endemic status in poultry. The HA of HPAIV H5N1 viruses circulating in Egypt encodes a multibasic amino acid motif (PQGERRR(KR)↓GLF) at the HA cleavage site, thus enhancing systemic spread of the virus in chickens (i.e., trachea, lung, brain, spleen, pancreas, liver, proventriculus, bursa of Fabricius and testis) and facilitating the bird-to-bird transmission of the virus [33]. In contrast, Egyptian H9N2 viruses isolated from a natural outbreak are all LPAIV, but their continuous shedding and spread by oral and cloacal routes without causing any clinical signs in infected poultry might cause undetected spread of the virus in the field [34]. Therefore, widespread co-circulation of H5N1 and H9N2 viruses has greatly raised a concern for potential generation of new sub- and geno-types of AIVs, thus making Egypt a potential epicenter for the next influenza pandemic and increasing concerns for public and animal health [35]. To address these concerns, this review has focused on the evolution and epidemiology of Egyptian H5N1 and H9N2 viruses in poultry, fields and live bird markets (LBMs); their zoonotic impact on human health; and preventive strategies for better control of viral infection and transmission.

## 2. Evolution of HPAI H5N1 Viruses in Poultry and Humans 

The evolutionary rate and positive selection of H5N1 viruses have increased due to vaccine-induced immune pressure in poultry or following adaptation upon transmission to new hosts (i.e., bird-to-human transmission) [36]. H5N1 viruses of clade 2.2 spread by migratory birds from South Eastern to Western Asia, Europe and Africa in 2005 and 2006 [37]. These viruses were detected in wild birds in Egypt in 2005 and subsequently isolated in poultry in February 2006 [38]. Since then, over 30 million birds have been killed by the virus or culled to control, thus causing serious losses in poultry production. Egypt has been declared as a country endemic to HPAI H5N1. The outbreaks of H5N1 viruses have prevalently occurred during winter, and since 2009, outbreaks have occurred during the summer and autumn, suggesting a change in the temporal pattern of the virus [39]. It is possible that the viruses circulating in Egypt have adapted to warmer environmental conditions [40]. 

The H5N1 viruses have rapidly evolved genetically and antigenically since the introduction of clade 2.2. viruses in Egypt, resulting in reclassification of the viruses into subclade 2.2.1 and 2.2.1.1 viruses (Table 1) [41,42]. The use of ineffective H5 vaccines in commercial poultry resulted in the emergence of a genetically- and antigenically-distinctive variant virus, clade 2.2.1.1 [43]. These variant strains harbor major changes in immunogenic epitopes of the HA protein, thus causing antigenic drift [44]. Five amino acid substitutions (at positions 74, 140, 141, 144 and 162) are primarily involved in the antigenic drift, and these mutations alone can reproduce the antigenic vaccine drift observed strains of clade 2.2.1.1 isolated from the field in 2008 [38]. The 2.2.1 and 2.2.1.1 viruses were mainly detected in backyard poultry and commercial poultry, respectively, from 2008–2011 [9,38,45]. Since 2012, the 2.2.1.1 viruses have rarely been detected, but the 2.2.1 viruses continued to evolve to form a new clade 2.2.1.2 [38]. The viruses have been isolated from non-vaccinated backyard birds, small-scale commercial poultry and humans since 2013 [46]. Some clade 2.2.1.2 viruses in 2014 possessed mutations R140K in antigenic site A and A86V in antigenic site E of the HA gene, indicating further antigenic drift. 

In recent years, Egypt has reported the highest number of human HPAI H5N1 cases (Table 3) [31]. Human H5N1 cases have often been caused by infection with clade 2.2.1 and 2.2.1.2 viruses, whereas clade 2.2.1.1 viruses hardly caused any human cases [47]. Furthermore, the clade 2.2.1.2 viruses contributed to a sharp increase in human H5N1 cases in 2014 and 2015. In 2016 and 2017, only Egypt reported human infection caused by clade 2.2.1.2 to WHO (Table 1). The clade 2.2.1 viruses that emerged between 2007 and 2008 had already acquired amino acid mutations (Q192H and the double 129Δ/I151T mutations), enabling human-type receptor binding during their transmission among the birds [45,48]. Subsequently, these changes have been found in H5N1 viruses isolated from humans, suggesting their attribution to efficient bird-to-human transmission. Other mutations (D94N, T156A, K189R and P235S) in the HA protein have also been shown to enhance binding affinity to the human-type receptor [49]. Most Egyptian H5N1 viruses possess two other mutations that may facilitate their transmissibility and replication in mammals: (1) the deletion in the 130 loop (129Δ) with a concurrent loss of glycosylation mutation (T156A) in the HA and (2) E627K mutation in the PB2 protein [50]. These mutations are critical for H5N1 virus transmissibility in ferrets and guinea pigs via respiratory droplets [45,51]. The loss of this glycosylation site located near the receptor-binding pocket has been shown to enhance the affinity of H5N1 virus binding to human-type receptors (SA2,6Gal) in ferret [52,53]. Interestingly, Egyptian H5N1 viruses have retained the classical avian α2,3 SA binding affinity. The Egyptian H5N1 viruses have not shown efficient transmissibility of respiratory droplets in ferrets [49]. Most human infections have resulted from direct exposure to H5N1 virus-infected poultry or poultry products, and human-to-human transmission has not been documented to date in Egypt. This suggests that currently circulating H5N1 viruses in Egypt lack enough alterations required for efficient human-to-human transmission. 

The NA protein is also an important surface protein by assisting the spread of viral infection [1]. Complementary evolution of the HA and the NA has been suggested to be essential for efficient virus replication, which requires the balanced actions of HA receptor-binding specificity and NA sialidase activity [54]. Analysis of the *NA* gene of Egyptian H5N1 viruses revealed that there was a 20-aa deletion in the NA stalk region (positions 49–68) [46]. Naturally-occurring avian viruses with shortened stalks are known to be virulent in poultry [55]. Most isolates of H5N1 viruses have not contained oseltamivir resistance markers at positions E119, H275, R293 and N295 (N1 numbering). However, a recent study has found one clade 2.2.1.1 virus (A/turkey/Egypt/7/2007) with a mutation at position N295S [38], which can be resistant to oseltamivir. 

In 2016, a newly-emerged H5N8 virus was isolated from wild birds (i.e., green-winged teal and common coots) in Egypt [56,57]. Phylogenetic analysis revealed that the genome of the Egyptian H5N8 viruses was related to recently characterized reassortant H5N8 viruses of clade 2.3.4.4 isolated from different Eurasian countries [56]. Further, chicken sera raised against commercial inactivated avian influenza-H5 vaccines did not react with these H5N8 viruses. This has complicated the epidemiological situation in Egypt where both HPAI H5N1 and LPAI H9N2 viruses are endemic among different poultry species. The establishment and dissemination of HPAI H5N8 across different bird species in Egypt would certainly make intricate the genetic diversity of AIV in Egypt and provide potentials for the emergence of reassortant strains with other subtypes. 

## 3. Widespread Circulation of H9N2 in Commercial and Backyard Poultry

In Egypt, H9N2 outbreaks have mainly occurred during the winter months, but sporadic outbreaks are observed year-round particularly in the Nile Delta. The majority of infected chickens showed respiratory distress [58,59] with a decrease in egg production in breeder and layer flocks [58]. In contrast, quails and some broiler flocks have also shown asymptomatic infections [60,61,62]. G1-like H9N2 viruses were first detected in poultry in 2010 and 2011. The emergence of H9N2 might have resulted from the importation of wild birds, legal or illegal trade of poultry, or other unidentified routes [62]. H9N2 viruses were widespread in commercial farm settings between 2009 and 2012 and have established endemic status in Egyptian poultry populations since 2012. The viruses have been isolated from a wide range of birds, including chickens, quails, ducks, turkeys and pigeons in commercial and backyard sectors [61]. The H9N2 strains circulating in Egypt between 2011 and 2015 mostly belonged to the G1 clade, similar to the circulating viruses in the Middle East with close phylogeny to the Israeli viruses. [63]. However, genetic analyses of all eight genes have revealed substantial genetic diversity and frequent reassortment events in internal genes of PB2, PA and NP among circulating Egyptian H9N2 strains (intrasubtype exchanges) [60]. A recent study further indicated that H9N2 virus isolated from domestic pigeons inherited five internal genes from Eurasian AIVs circulating in wild birds [64]. 

Egyptian H9N2 viruses possessed several genetic markers of increased transmission to mammalian hosts [65]. In 2010, H9N2 viruses carrying HA L234 (H9 numbering) were detected in poultry [59,61]. Further, H9N2 isolates from 2011–2015 had H191 and L234 (H183 and L226, respectively, with H3 numbering), which correlate with a shift in affinity of the HA from the avian-type receptor towards the human-type receptor [62,63,64]. However, markers of mammalian adaption in the PB2 protein (K627 and N701) have not been found in the H9N2 isolates [62,63].

H9N2 viruses phylogenetically group into three clusters [1,2,3] within subclade B of the G1 clade. Antigenic analysis has shown a close clustering of the Egyptian H9N2 viruses with other recent G1-B-like H9N2 strains [66]. In contrast, the viruses have shown a significant antigenic distance from viruses outside the G1-B subclade. Further antigenic analysis of H9N2 viruses isolated from 2011–2015 showed antigenic conservation of different Egyptian H9N2 isolates from chickens, pigeons and ducks [62,64]. However, antigenic drift of H9N2 isolated from quails has been found as a result of mutations in the antigenic sites A (N166D) and B (D153N and N201A), suggesting that the virus can be a new escape mutant variant cluster with an adaptive evolution in quails [67,68]. 

## 4. Potential Reassortment of AIVs

Reassortment plays an important role in the evolution of segmented AIVs [1]. Frequent reassortment events between HPAI H5N1 and H9N2 have been reported in Bangladesh [59], Pakistan [24], and China [25]. In particular, H9N2 has contributed to generating highly zoonotic AIV in China by donating their six internal genes [23,26,27]. Therefore, surveillance of reassortant viruses in the field can be critical for public health. In Egypt, the existence of reassortment between the Egyptian A/H5N1 clades and H9N2 has not been detected in the field [49,60,63,64,69,70,71]. The lack of detection of reassortant viruses may have been due to insufficient surveillance for circulating viruses. In addition, HPAI H5N1 and H9N2 may have circulated in mutually exclusive host populations or may have been separated by geographical or temporal barriers. For example, the presence of the H5N1 2.2.1.1 clade circulated from 2008–2011 was highly restricted to industrial poultry farms. Thus, divergent evolution was fostered even by the sectoral separation of poultry populations in Egypt. 

Although genetic incompatibility between the two viruses could have resulted in a lack of reassortment event, a recent study demonstrated generation of reassortants with various genotypes by co-infecting Egyptian H5N1 clade 2.2.1.2 and H9N2 viruses in embryonated chicken eggs [72]. The viruses were coamplified in embryonated chicken eggs and then plaque-purified for sequencing analysis of selected clones. Reassortants were restricted to the H5N1 subtype and acquired from two to all six of the internal segments of the H9N2 virus. Replication of the reassortant viruses in ferret did not indicate enhanced zoonotic potential. Despite the potentials of generating reassortant viruses, their spread would be determined by their fitness to outcompete the parental viruses in the field. Furthermore, enhanced surveillance and continuous examination of the whole genome of both subtypes are required to improve the early detection of reassortant viruses.

## 5. Epidemiology of Zoonotic H5N1 and H9N2 Viruses in Egypt 

The spread and outbreaks of AIV have increased as a result of global trade, increase poultry production, climate changes, bird migration, human movement and increasing global population [70]. Egypt lies at the crossroads of two major spatially-overlapping migration flyways: the Black Sea-Mediterranean flyway and the East African-West Asian flyway linking Africa, Europe and Asia [35]. The Nile Delta is an important wintering stopover for millions of birds between Eurasia and Africa, where birds from different origins mix and mingle. The distribution of infected flocks along the main migration routes of birds in Egypt has suggested that the virus might be introduced via wild birds [73]. The high density of poultry and human populations along the Nile valley enables frequent and close contacts between poultry, live-stock and humans. 

Poultry farming in Egypt includes commercial farming with a high density of poultry population, free-range rearing (backyards, rooftops) and trading via LBMs [57]. Egyptian families raise poultry traditionally in a backyard setting, accounting for approximately a third of poultry production in the country [74]. Backyard birds have become an intermediate transmission host for AIV infection of poultry in the commercial sector and LBMs and for human infection. In general, apparently healthy birds are usually sold in traditional LBM's, but approximately 38% of households slaughter their birds once they become ill. In addition, backyard farms have outdoor enclosures with low biosecurity, whereas commercial farms have high biosecurity by limiting poultry contact with potentially infected wild birds. In Egypt, due to insufficient slaughtering facilities, trading of freshly-slaughtered poultry meat is mainly conducted in LBMs [39]. In fact, LBMs have been implicated in numerous outbreaks of AIV worldwide [75,76]. LBMs play an important role in the dynamics of AIV transmission and evolution, thus facilitating reassortment between viruses. In a surveillance, H5N1 was detected from 0.1% of examined commercial poultry farms, 10.5% of backyard birds and 11.4% of LBMs [40]. Similarly, a surveillance study conducted in Qena and Luxor has shown that infection rates for backyard birds and birds from local bird markets (86.84% and 76%) was higher than those in birds from commercial farms (13.16% and 24%), respectively [77]. In general, bird-to-human transmission has occurred due to contact and/or slaughtering and defeathering of infected backyard birds [78]. Specifically, the cases of human infections have been reported by exposure to backyard poultry (70%), bred domestic birds (26%), slaughtered poultry (14%) or dead birds (4%) [78]. The main symptoms were fever (98%), sore throat (94%) and cough (83%). 

Interestingly, prevalence of H5N1 has been found in backyards raising chickens and waterfowl together in the same vicinity and LBMs having chickens and waterfowl together [40]. In contrast to chickens, waterfowl can be silently infected with H5N1. Particularly, the infected ducks and geese may play a role as a reservoir and/or source for the transmission of H5N1 to chickens and may prolong the circulation of the virus in LBMs [40]. Mallard ducks experimentally infected with H5N1 have been shown to excrete the virus asymptomatically for 17 days [79]. The Food and Agriculture Organization (FAO) has also established that ducks and geese pose a very high risk of infection to households and small-scale poultry farms [80]. Therefore, it will be important to understand the local circulation of AIV in domestic poultry and wild birds in Egypt to identify the risk factors in designing and implementing effective control strategies. 

Sero-surveillance data indicated occupational exposure of humans to both H5N1 and H9N2 in Egypt [65]. In Egypt, most reported cases of HPAI H5N1 infections have affected backyard poultry producers with a case-fatality rate of 36% [65]. Most cases of human infections have been reported during eight of the 12 months with the highest incidents in January and February, reflecting a seasonal pattern conforming to the climate, with influenza activity peaks in the colder months [81]. The seroprevalence rate of antibodies against H5N1 (titers > 80) was 2%. H9N2 viruses are considered a pandemic risk, given their endemicity, ability to infect numerous species and a lack of population-wide immunity. In 2015, the first human H9N2 case was reported from Egypt [63]. In 2015, three children with a history of exposure to poultry were found positive for the RNA of H9N2. Human infections showed transient influenza-like illness, but subsided without sequelae. In humans, the seroprevalence of antibodies against H9N2 virus ranged between 1.2% and 9% [65]. Due to the potential risk, the World Health Organization monitors the continual evolution of H5N1 and H9N2 clades to provide candidate vaccine viruses for public health purposes [82].

The viruses have sporadically infected other mammalian species. Specifically, pigs have the largest epidemiological role in the evolution of new influenza viruses, since they are susceptible to infection with both avian and human influenza viruses [83]. In Egypt, exposure of pigs to influenza viruses has also been reported [65,84]. Serological analyses showed the presence of neutralizing antibodies against H9N2, H5N1 and human pandemic H1N1 viruses in pigs sampled in Cairo, Egypt, where most of the swine population is located. Molecular detection of the viruses further confirmed the infection of pigs with avian and human viruses in abattoir pigs, suggesting that the routine surveillance of AI in pigs will be required to monitor the emergence of reassortant viruses and their pandemic potential [64,84]. 

## 6. Challenges with Current Vaccination Strategy in Egypt

Vaccination can be an efficient strategy to control AIV [13]. Since the emergence of HPAI H5N1, Egypt has vaccinated poultry as a national strategy to control HPAI H5N1 and H9N2. Despite nationwide attempts of eradication, the viruses have gained endemic status in poultry in Egypt and continued to threaten poultry production and public health [55]. The goal of national vaccination campaigns to achieve population immunity has been difficult to achieve because of limitations in financial and human resources. For example, in 2006, the vaccine coverage rate for H5N1 was 69.9% [85]. The exact population of household poultry is unknown; however, an estimated 4–9.5 million households raise poultry in confined spaces in houses [76]. Vaccination in village poultry has been a challenge and hindered achieving a population immunity with actual vaccination coverage rates between 27.8% and 48.6%. 

Most commonly-used vaccines are inactivated, oil-emulsified, whole AIV vaccines. At least 24 commercial inactivated avian influenza H5 vaccines have been licensed for use at poultry farms in Egypt [86]. Vaccine seed strains include classical H5 lineage viruses and reverse genetics-engineered reassortant viruses containing H5N1 virus *HA* and *NA* genes and the remaining genes from A/Puerto Rico/8/1934 (H1N1). However, the genetic dissimilarity between commercial vaccines and currently circulating viruses has resulted in inefficient vaccination in the field. These vaccines confer suboptimal protection of poultry that can result in continuous circulation of AIV in the field. This further leads to vaccine-induced escape mutants as shown with the emergence of the antigenic drift variant (clade 2.2.1.1) in commercial poultry. In addition, administration of vaccines, which requires handling and injection of individual birds, is labor intensive. Particularly, administration of prime-and-boost vaccination rounds is challenging with numerous birds in industrial settings and rapid turnover rates of poultry populations [87]. This is not a practical vaccination approach for village farmers. For the vaccination of broiler poultry, a long withdrawal period for inactivated vaccines has also limited AIV vaccination in hatcheries. Indeed, the use of one-day-old age vaccination in broilers using inactivated vaccines resulted in a failure of vaccination by inducing inadequate immune responses, even further decreasing the effective immunity in the population [88]. 

In the field, the infection of H9N2 viruses in commercial poultry can also induce cross immunity against H5N1, which can hinder efficient vaccination and control of H5N1 [60,89]. This has been demonstrated by evaluating the impact of H9N2 infection on the course of consecutive infection with a lethal Egyptian HPAI H5N1 [90]. Chickens previously infected with an Egyptian LPAI H9N2 developed a delayed course of infection, with prolonged viral shedding following challenge with a lethal dose of Egyptian HPAI H5N1 clade 2.2.1.2 H9N2 pre-infection modulated, but did not conceal clinical disease by HPAI H5N1. In contrast, homologous H5 vaccination abolished clinical syndromic surveillance, although vaccinated clinically healthy birds were capable of shedding and spreading the virus. Cross-reactive cellular immunity induced by the internal proteins of the H9N2 virus plays a critical role in the delayed development of clinical signs and decreased virus excretion [89,91]. In Egypt, culling is only conducted when clinical disease is evident [89]. Therefore, this can be a drawback for efficient control of AIV and needs to be considered in developing efficient vaccines against HPAI H5N1. 

Backyard chickens are being raised in a low biosecurity setting, thus facilitating the transmission of viruses [76]. However, vaccination has not been implemented for backyard chickens, and the efficacy of vaccines for backyard poultry has not been well evaluated. In a backyard setting, double doses of H5N1 vaccination based on a contemporary Egyptian clade 2.2.1.2 virus has been shown to induce immune responses in turkeys, ducks, geese and chickens raised together [64]. When challenged, vaccinated birds survived and shed less virus in comparison with unvaccinated birds. However, unvaccinated ducks showed no symptoms of infection and survived the duration of the experiment, indicating a low sensitivity of Pekin ducks to currently circulating 2.2.1.2 H5N1 challenge virus. Moreover, vaccinated ducks shed more virus as compared to vaccinated birds of other species. This study demonstrates the importance of using a genetically-matched vaccine with a currently circulating strain to efficiently protect chickens in a backyard setting. Since the protective efficacy in the tested poultry was variable, mixing various species in the backyards can enhance interspecies transmission of AIV. In fact, Egypt has many “Trojan horses” in the epidemiology of AIV including asymptomatic vaccinated birds, such as subclinically-infected ducks, geese and other waterfowl. Since in most cases, biosecurity measures are impractical or impossible to implement and enforce, vaccination is one of the few preventive strategies available to protect domestic ducks against H5N1 [88]. However, vaccine coverage rates of meat ducks (15%) are relatively low compared to high vaccine coverage rates of meat (78.2%) and layer (31.6%) chickens [85]. Ducks have also been known to require higher doses of vaccination than chickens for adequate immunity for protection from HPAI H5N1 [90]. Further, the success of the vaccination program can be affected by the duck species and/or breed, vaccination protocols (number of doses, ages of birds) and efficacy of vaccines [88]. Therefore, it will be advantageous to develop a comprehensive vaccination strategy including domestic waterfowl and to develop effective vaccines for domestic waterfowl. 

## 7. Enhancing Preventive Strategies for Efficient Control of AIV

Eradication of AIV was unsuccessful in Egypt despite the vaccination of poultry immediately after the introduction of HPAI H5N1. The prolonged survival and shedding of AIV in infected chickens have fostered the endemic status of the virus and have caused antigenic drift. To enhance the protective efficacy of vaccines and efficient vaccination in the field, development of a new platform of vaccine will be desirable for cost-effective and massive vaccination of short-lived poultry. Currently, use of live attenuated influenza vaccines in poultry is not recommended by the World Organisation for Animal Health (OIE) due to the potential risk of reassortment or mutations for generating AIV [92]. Alternatively, replicating viral vector vaccines offer a live vaccine approach without requiring the involvement of complete pathogens. To facilitate the administration of vaccines (mass vaccination), live-attenuated avian virus (i.e., Newcastle disease virus, fowlpox virus and turkey herpesvirus) vectored vaccines have been licensed for poultry vaccination with limited use (<5% of usage) in China and in Mexico [92,93,94,95,96]. Despite the advantage of mass vaccination (i.e., drinking water and spray), maternal antibodies to vaccine vectors have hampered the efficacy of vaccines in field vaccination [13]. Currently, there is a limited vaccination approach for overcoming the interference by maternal antibodies in poultry within the first two weeks of life, which can be particularly critical for the protection of broiler chickens from HPAI H5N1. 

Recently, a new vaccine vector platform using the chimeric Newcastle disease virus (NDV)-vectored vaccine has been developed for vaccination of poultry [97]. In contrast to other strategies using the conventional NDV vector [98], the chimeric NDV vector was constructed by replacing the major antigens of ectodomains of F and HN proteins with those of avian paramyxovirus serotype-2 (APMV-2). The chimeric NDV is serologically distant from NDV, suggesting that maternal antibody to NDV could not inhibit the replication of chimeric vaccine virus. One-day-old commercial chickens were vaccinated by a chimeric NDV-vectored vaccine for inducing early immunity and subsequently boosted with a conventional NDV vectored vaccine for inducing robust and long-lasting immunity [99]. This heterologous prime-boost vaccination strategy can provide improved immunization with a convenient platform for mass vaccination in the field. Since NDV is also co-circulating with HPAI H5N1 in poultry in Egypt [100,101], boosting with LaSota-vectored vaccine can also serve as a dual vaccination approach, thus making it economical and effective for the poultry farmers. This system can be used as a “differentiating infected from vaccinated animals” (DIVA) vaccine, thus facilitating epidemiological surveillance and vaccination systems for AIV in the field. Furthermore, vaccine viruses can be readily engineered by placing protective antigens of H5N1 or H9N2and by further updating the antigens with a replaceable vaccine cassette of currently-circulating virus in the field. This will make it feasible to apply this vaccination system for the generation of tailored vaccines targeting the Egyptian strains of AIV. Indeed, to maintain the most effective vaccination program, the field virus should be monitored for antigenic changes, and the vaccine should be tested against new variants or at a minimum the vaccine should be re-evaluated every 2–3 years for protection against currently circulating field viruses [88]. If necessary, vaccine seed update can be a strategic approach to provide protection of poultry against continuously emerging H5N1 strains. Even H9N2 viruses have diverged into different sublineages, although Egyptian H9N2 viruses share a close genetic relationship. Recently, H9N2 isolates from quail have shown antigenic drift [67,68]. The antigenic relatedness of different sublineages needs further examination to select suitable vaccine strains. 

In Egypt, vaccination of poultry has been used as the front line preventive strategy to prevent AI infection. Current endemic status of H5N1 and H9N2 viruses in the field implies the need for a comprehensive preventive strategy with the combination of effective vaccination, biosecurity, education, surveillance, rapid diagnosis and depopulation of affected flocks [13,85]. Poultry in backyards and LBMs is a major source of the continuous outbreaks in Egypt. It will be necessary to continuously educate famers about biosecurity and the increasing risk of open-range poultry [76]. In general, voluntary culling of infected backyard birds is limited, thus making it difficult to eradicate the disease. Vaccination of poultry may not be feasible for backyard poultry growers in Egypt. Since they commonly purchase different types of birds at 3–4 weeks of age from specific nursery farms, intervention of effective vaccination of young birds at nursery farms may be a practical strategy [64]. The poultry meat trade in Egypt relies strongly on LBMs, partly because of consumers’ long-standing cultural preference for fresh poultry meat [39]. All categories (big, small, daily and weekly) of LBMs in Egypt operate with minimal to no biosecurity standards, and veterinary inspections are rarely implemented. In LBMs, major risk factors associated with biosecurity compliance are wild animals traded in the market and the lack of mandatory routine disinfection and hand washing after slaughter, as well as limited fencing and gates around the LBMs, suggesting that biosecurity and compliance level must be monitored and enforced [102]. It can also be beneficial to reduce inter-governorate inter-regional movements associated with poultry trade through promotion of regional trade to prevent the spread of AIV [76]. The Food and Agricultural Organization of the United Nations has also recommended implementing a policy of no carryover of animals (i.e., housing the same animals in the marketplace for multiple days), which improves live-market design to reduce AIV exchange [103]. The One Health concept focuses on the relationship and interconnectedness between humans, animals and the environment and recognizes that the health and well-being of humans is intimately connected to the health of animals and their environment. Both human and poultry populations will continue to rise in Egypt. Therefore, continued co-circulation of H5N1 and H9N2 in the field and environment can increase the occurrences of AI outbreaks in poultry and humans [41]. In fact, despite declines in other countries, AIV continues to circulate and enter the human population in Egypt. The epidemiology of AIV also implies the need for enhanced surveillance and monitoring of AIVs in Egypt for early detection of human-transmissible AIVs. Established genetic analysis of AIVs to identify their human transmissibility can be utilized to develop a rapid and feasible intervention strategy for efficient control of emerging viruses.

## 8. Conclusions

Egyptian H5N1 and H9N2 viruses have evolved to possess molecular markers that can potentially enhance the transmissibility of the viruses from birds to humans. A practical strategy for rapid, efficient and economical immunization of poultry in the field will be required for a better control of zoonotic avian influenza viruses for animal health and public health. As a preventive strategy, education of farmers for effective vaccination and enhancing biosecurity measures can also be beneficial in reducing the circulation of AIVs in the field. Co-circulation of zoonotic H5N1 and H9N2 viruses in poultry and the emergence of H5N8 in wild birds has complicated the epidemiological situation in Egypt. As a preventive strategy, surveillance efforts can be extended to monitor the evolution of AIVs and the emergence of human transmissible viruses in wild aquatic birds and domestic poultry in commercial farms, backyards and LBMs. 

## Figures and Tables

**Table 1:** Evolution of the Gs/GD lineage of the highly pathogenic avian influenza (HPAI) H5 subtype.

Clade	Host	Country
1.1.2	Poultry, human *	Cambodia
	Poultry, human	Viet Nam
2.1.3.2	Poultry, human	Indonesia
2.2.1	Poultry	Libya
	Poultry, feline	Israel
	Poultry, human	Egypt
2.2.1.1	Poultry	Egypt
2.2.1.2	Poultry, human	Egypt
2.3.2.1	Poultry, human	Bangladesh
	Poultry	Bhutan
	Human	Cambodia
	Poultry, wild bird, human	China
	Wild birds, poultry	India
	Poultry, wild birds	Nepal
	Poultry	Viet Nam
2.3.4.4 (H5N1/N6)	Poultry, wild birds	China
2.3.4.4 (H5N6)	Poultry	Lao PDR
	Poultry	Viet Nam
	Poultry, wild birds	South Korea
	Poultry, wild birds	Japan
2.3.4.4 (H5N8)	Poultry, wild birds	Germany
	Poultry, wild birds	Netherlands
	Poultry, wild birds	UK
2.3.4.4 (H5N2/N8)	Poultry, wild birds	US
	Poultry, wild birds	Canada
2.3.4.6 (H5N1/N6/N8)	Poultry, human	China
2.3.4.6 (H5N8)	Poultry	Japan
	Poultry, wild birds	South Korea
7.2	Poultry	China

* Human cases of H5N1 infection reported to WHO.

**Table 2:** Clades of H9N2 viruses isolated from poultry.

Clade	Prototype Strain	Regions
G1-like	A/quail/Hong Kong/G1/97	China, Bangladesh, Pakistan, India, Middle East, Egypt
Y280-like	A/duck/Hong Kong/Y280/97	China
G9-like	A/chicken/Hong Kong/G9/97	China
BJ94-like	A/chicken/Beijing/1/94	China
Y439-like	A/duck/Hong Kong/Y439/97	South Korea
Korean-like	A/chicken/Korea/38349-p96323/96	South Korea

**Table 3:** Cumulative number of confirmed human cases for avian influenza A(H5N1) reported to WHO, 2003–2017.

Country	2003–2009		2010–2014		2015		2016		2017		Total	
	Cases	Deaths	Cases	Deaths	Cases	Deaths	Cases	Deaths	Cases	Deaths	Cases	Deaths
Cambodia	9	7	47	30	0	0	0	0	0	0	56	37
China	38	25	9	5	6	1	0	0	0	0	53	31
Egypt	90	27	120	50	136	39	10	3	3	1	359	120
Indonesia	162	134	35	31	2	2	0	0	1	1	200	168
Viet Nam	112	57	15	7	00	0	0	0	0	0	127	64
